# A Combined Phytochemistry and Network Pharmacology Approach to Reveal the Potential Antitumor Effective Substances and Mechanism of *Phellinus igniarius*

**DOI:** 10.3389/fphar.2019.00266

**Published:** 2019-03-19

**Authors:** Yu Dong, Ping Qiu, Rui Zhu, Lisha Zhao, Pinghu Zhang, Yiqi Wang, Changyu Li, Kequn Chai, Dan Shou, Huajun Zhao

**Affiliations:** ^1^Department of Medicine, Zhejiang Academy of Traditional Chinese Medicine, Hangzhou, China; ^2^College of Pharmaceutical Sciences, Zhejiang Chinese Medical University, Hangzhou, China; ^3^Institute of Translational Medicine and Jiangsu Key Laboratory of Integrated Traditional Chinese and Western Medicine for Prevention and Treatment of Senile Diseases, Medical College, Yangzhou University, Yangzhou, China; ^4^Zhejiang Key Laboratory of Tumor Diagnosis and Treatment with Integrated TCM and Western Medicine, Hangzhou, China

**Keywords:** *Phellinus igniarius*, antitumor, phytochemistry, network pharmacology, effective substances, mitochondrial apoptosis pathway

## Abstract

*Phellinus igniarius* (*P. igniarius*) is a medicinal fungus that is widely used in East Asia for the adjuvant treatment of cancer. To elucidate the antitumor effective substances and mechanism of *P. igniarius*, we designed an approach incorporating cytotoxicity screening, phytochemical analysis, network pharmacology construction, and cellular and molecular experiments. The dichloromethane extract of *P. igniarius* (DCMPI) was identified as the active portion in HT-29 cells. Nineteen constituents were identified, and 5 were quantified by UPLC-ESI-Q/TOF-MS. Eight ingredients were obtained in the network pharmacology study. In total, 473 putative targets associated with DCMPI and 350 putative targets related to colon cancer were derived from online databases and target prediction tools. Protein-protein interaction networks of drug and disease putative targets were constructed, and 84 candidate targets were identified based on topological features. Pathway enrichment analysis showed that the candidate targets were mostly related to reactive oxygen species (ROS) metabolic processes and intrinsic apoptotic pathways. Then, a cellular experiment was used to validate the drug-target mechanisms predicted by the system pharmacology analysis. Experimental results showed that DCMPI increased intracellular ROS levels and induced HT-29 cell apoptosis. Molecular biology experiments indicated that DCMPI not only increased Bax and Bad protein expression and promoted PARP and caspase-3/9 cleavage but also down-regulated Bcl-2 and Bcl-xl protein levels to induce apoptosis in HT-29 cells. In conclusion, our study provides knowledge on the chemical composition and antitumor mechanism of *P. igniarius*, which may be exploited as a promising therapeutic option for colon cancer.

## Introduction

Cancer serves as a major public health problem worldwide and is expected to surpass heart disease as the leading cause of death in the next few years (Siegel et al., 2016). In the past decade, numerous significant advances in cancer research have revealed the genetics and pathologies of malignant tumors, which, in turn, facilitates the development of novel anticancer agents (Hare et al., 2017). Interestingly, multiple bioactive phytochemicals in consumed edible and medicinal fungi have recently attracted considerable attention as potential candidates for anticancer agents (Li et al., 2015).

*Phellinus igniarius* (*P. igniarius*), a well-known mushroom belonging to the genus *Phellinus* in the polyporaceae family, is a physiologically functional food and exemplary source of natural medicine that has been widely used in China, Japan, Korea, and other countries (Ma et al., 2016). *P. igniarius* possesses high anti-inflammatory, antioxidant and antitumor biological activities due to its accumulation of various secondary metabolites, including polysaccharides, flavonoids, polyphenols, steroids and organic acids (Dong et al., 2015; Shou et al., 2016). Notably, recent studies have creatively focused on the potential functions of *P. igniarius* extracts and their constituent compounds in the prevention and treatment of cancer (Zhou et al., 2014; Sangdee et al., 2017). A considerable amount of evidence indicates that *P. igniarius* extracts resulting from water, alcohol, ethyl acetate and other solvent extractions have a significant inhibitory effect on various tumor cells, such as S180, PC3, SK-HEP-1, and HT-29 cells (Yang et al., 2006; Song et al., 2008; Jeon et al., 2013). To date, no prior system reports on the chemical composition of *P. igniarius* contribute to its antitumor activity or functional mechanisms.

Network pharmacology is now popularly utilized to discover the basis of pharmacodynamic substances, explore their molecular mechanisms of action, and elucidate their scientific connotations (Kim et al., 2017). Especially, TCM network pharmacology focuses on the wholeness and systemicity of the interactions between components, targets and diseases of TCM (Cao et al., 2018; Huang et al., 2018), and is crucial to select the beneficial therapeutic targets of TCM, typical TCM syndromes and corresponding classic formulas (Zhang et al., 2017). At the same time, TCM network pharmacology substantially reduces the workload of follow-up experimental studies on TCM.

In the present study, we designed an approach incorporating cytotoxicity screening, phytochemical analysis, network pharmacology construction, and cellular and molecular biology validation to clarify the antitumor effective substance and mechanism of *P. igniarius*. Notably, this is the first integral study using multiple methods in combination to elucidate the antitumor efficacy substances and mechanism of this large medicinal fungus.

## Materials and Methods

### Chemicals and Materials

*Phellinus igniarius* sporocarps were purchased from Zhejiang Qingzheng Biotechnology Co. Ltd. (Hangzhou, China). Reference standards (purity > 98%) of protocatechuic aldehyde (671E-QHX2) and osmundacetone (RC5E-FH31) were purchased from the National Institute for the Control of Pharmaceutical and Biological Products (Beijing, China), and naringenin (170124), eriodictycol (170309) and sakuranetin (170124) were purchased from Beijing Century Aoke Biology Research Co., Ltd. (Beijing, China). HPLC grade methanol and acetonitrile were purchased from Merck (Darmstadt, Germany). Distilled water was purchased from Watson’s Food & Beverage Co., Ltd. (Guangzhou, China). Leucine enkephalin and formic acid were purchased from Sigma-Aldrich (Darmstadt, Germany).

Human hepatoma carcinoma (HepG2, SMMC7721), human gastric carcinoma (BGC-823, SGC790, AGS), human colon carcinoma (HT-29) and human lung carcinoma (A549) cells were purchased from American Type Culture Collection (Rockefeller, MD, United States). DMEM and RPMI 1640 cell culture mediums were purchased from HyClone Corporation (Logan, UT, United States). Fetal bovine serum was purchased from Gibco Corporation (Grand Island, NE, United States). MTT was purchased from Sigma-Aldrich (Darmstadt, Germany). An AnnexinV-FITC/PI apoptosis detection kit was purchased from BD Biosciences (Franklin lakes, NJ, United States). A ROSs assay kit and JC-1 dye were purchased from Beyotime Biosciences (Nanjing, China). Antibodies against PARP (#9532), Caspase-3 (#9662), Caspase-8 (#6790), Caspase-9 (#9508), Bax (#5023), Bcl-2 (#2870), Bcl-xl (#2764), and β-actin (#4970) were purchased from Cell Signaling Technology (Boston, MA, United States). Antibody against Bad (ab32445) was purchased from Abcam (Cambridge, MA, United States). HRP-conjugated secondary antibody was purchased from Bio-Rad (Hercules, CA, United States).

### *P. igniarius* Extract Preparation

The dried *P. igniarius* sporocarps were crushed into a powder (with an approximately 100 mesh screen) using a plant disintegrator. In total, 600 g of powder was weighed, immersed in 3 L of 95% (v/v) ethanol for 30 min, and extracted in an ultrasonic bath 3 times for 1 h each time. The extracted solution was merged and evaporated by a rotary evaporator, and distilled water was added to 1 L of the suspension (containing 20% ethanol). The suspension was then subjected to liquid-liquid extraction with petroleum ether, dichloromethane, ethyl acetate and *n*-butanol successively. Each extract fraction was merged, evaporated and then freeze-dried to obtain the petroleum ether extract (0.32 g), dichloromethane extract (0.51 g), ethyl acetate extract (2.23 g), and *n*-butanol extract (1.28 g).

### Cell Culture

Human hepatoma carcinoma (HepG2) and human gastric carcinoma (BGC-823) cells were cultured in DMEM. Human gastric carcinoma (SGC790, AGS), human colon carcinoma (HT-29) and human lung carcinoma (A549) cells were maintained in RPMI 1640 medium. Human hepatoma carcinoma cells (SMMC7721) were cultured in DMEM/F12 medium. Then, the three media were supplemented with 10% fetal bovine serum, 100 units/mL penicillin and 100 μg/mL streptomycin. The cultures were maintained in a humidified incubator containing 5% (v/v) CO_2_ at 37°C.

### Cytotoxicity Experiment

The cytotoxic effects of the *P. igniarius* extracts were detected using the MTT calorimetric method. Briefly, cells (3 × 10^3^ cells/well) were seeded in 96-well plates and incubated for 24 h. The supernatant was subsequently aspirated, and the cells were exposed to various concentrations of extracts and 0.05% DMSO (v/v). After 72 h of incubation, MTT solution (0.5 mg/mL) was added to each well and incubated for 4 h. Thereafter, the medium containing MTT was removed and replaced with 120 μL of DMSO to dissolve the formazan crystals. Next, the plates were shaken for 20 min, and the absorbance at 570 nm was recorded using an enzyme-linked immunosorbent assay reader (TECAN, Switzerland).

In addition, HT-29 cells (5 × 10^5^ cells/well) were seeded into a six-well plate and incubated for 24 h. Then, the medium was replaced with RPMI-1640 medium containing different concentrations of dichloromethane extract of *P. igniarius* (DCMPI). After incubation for 72 h, the cytomorphology of the cells was observed and digitally photographed using a phase contrast microscope (Olympus, Japan).

### Chemical Profile Analysis by UPLC-ESI-Q/TOF-MS

The UPLC-ESI-Q/TOF-MS system consisted of an Acquity^TM^ ultra performance liquid chromatography (UPLC) system (Waters Corporation, Milford, MA, United States) and a Synapt G2 mass spectrometer (MS) (Waters MS-Technologies, Manchester, United Kingdom) equipped with an electrospray ion (ESI) source. The system and data were controlled by MassLynx (V4.1) software. Chromatography was performed on an Acquity UPLC BEH C_18_ column (2.1 × 150 mm, 1.7 μm, Waters Corporation, Milford, MA, United States) at a flow rate of 0.3 mL/min and a 40°C column temperature. The optimal mobile phases consisted of A (HCOOH: CH_3_CN = 0.1: 100, v/v) and B (HCOOH: H_2_O = 0.1: 100, v/v): 0–1 min, 1–8% A; 1–2.5 min, 8–15% A; 2.5–4.5 min, 15–20% A; 4.5–5.5 min, maintained at 20% A; 5.5–7.5 min, 20–30% A; 7.5–13 min, 30–60%; 13–14 min, 60–99% A; and 14–15 min, maintained at 99% A. The extracts were dissolved in methanol to a concentration of 1 mg/mL and filtered through a 0.22 μm membrane. A 2 μL aliquot of sample solution was injected for analysis.

The full-scan LC-MS data were acquired in both positive and negative ion modes from 50 to 1500 Da with a 0.3 s scan time. The optimal Q/TOF-MS conditions were as follows: capillary voltage 3.0 kV, sampling cone voltage 40.0 V and extraction cone voltage 5.0 V for positive ion mode; capillary voltage 2.4 kV, sampling cone voltage 35.0 V and extraction cone voltage 3.5 V for negative ion mode. The source temperature and desolvation gas temperature were set to 150 and 350°C, respectively, and the cone gas flow and desolvation gas flow were set to 60 L/h and 550 L/h, respectively. The collision energy was set to 4 eV for positive ion mode and 2 eV for negative ion mode. Sodium formate solution was used to calibrate the mass spectrometer prior to the experiment. Leucine-enkephalin was used as an external reference (Lock-Spray^TM^) to correct the mass during data acquisition via a LockSpray interface, generating reference ions at *m/z* 556.2771 Da ([M+H]^+^) and *m/z* 554.2615 Da ([M-H]^−^) in the positive and negative ion modes, respectively.

### Constituent Identification

All LC-MS and MS/MS data were processed with MassLynx^TM^ (V4.1) software. Molecular formula speculations of the compounds were determined with Elemental Composition software. Structural identification of the main compounds, which included the chemical structure, accurate molecular mass and potential molecular fragmentation pathways, was determined with Mass Fragment software. Previously published compounds were identified by comprehensively searching databases, such as PubMed^1^, Chemspider^2^, HMDB^3^, and Metlin^4^. Compounds with standard materials were validated using reference standards.

### Main Ingredient Quantification for DCMPI

DCMPI was dissolved in methanol to a concentration of 1 mg/mL as a sample solution. Mother stock solutions (approximately 1 mg/mL) of protocatechuic aldehyde, osmundacetone, eriodictyol, naringenin and sakuranetin were separately prepared in MeOH. Further, combined spiking stock solutions of the five reference substances were prepared in MeOH from the mother stock solutions by stepwise dilution in the ranges of 4.61–73.15 μg/mL for protocatechuic aldehyde, 3.97–63.44 μg/mL for osmundacetone, 3.91–62.50 μg/mL for eriodictyol, 4.20–67.19 μg/mL for naringenin and 4.04–64.69 μg/mL for sakuranetin. The sample solution and each combined spiking stock solution were filtered through a 0.22 μm membrane, and a 2 μL volume was injected into the UPLC-ESI-Q/TOF-MS system for analysis. The LC-MS conditions were the same as those previously described.

### Prediction of Drug Targets for DCMPI

Eight major DCMPI components, including five validated components (protocatechuic aldehyde, osmundacetone, eriodictyol, naringenin and sakuranetin) and three identified components (inoscavin A, phelligrin A and phelligrin B) with a peak area greater than 5% were included in the network pharmacology study. By searching for the relevant predictive genes of the 8 main components at the websites PharmMapper Server^5^, SEA Search Server^6^, STITCH^7^ and TCMSP^8^, the DCMPI prediction genes were obtained.

### Collection of Predicted Targets Related to Colon Cancer

Two main methods were used to identify colon cancer-related targets. To identify the main DEGs between normal human colon samples and colon cancer samples, GDS4382 microarray data were downloaded from the GEO^9^. The data set consisted of 34 human samples; GEO analysis was performed using 17 normal colon samples and 17 colon cancer samples. DEGs were defined by the Bioconductor/R limma package. *P* < 0.05 and a fold change ≥ 2 were applied. The known targets associated with colon cancer were derived from five databases, GAD, OMIM^10^, TTD^11^, DrugBank^12^ and CoolGen^13^, using ‘colon cancer’ as the key word.

### Construction of the PPI Network

Protein–protein interaction data from the six currently available PPI databases, the Human Protein Reference Database, the Biomolecular Interaction Network Database, the Biological General Repository for Interaction Datasets, the Database of Interacting Proteins and the Molecular INTeraction Database, were searched using the Cytoscape plugin BisoGenet. An interactive network of DCMPI drug targets and colon cancer-related targets was constructed based on interaction data, and the network was visualized utilizing Cytoscape software (version 3.2.1).

### Definition of Network Topology Feature Set

By calculating the ‘betweenness centrality,’ ‘degree centrality,’ ‘eigenvector centrality,’ ‘closeness centrality,’ ‘network centrality’ and ‘local average connectivity’ using CytoNCA, the topological nature of each node in the interactive network was analyzed. The definitions and calculation formulas of these six parameters represent the topological importance of the nodes in the network, with more important nodes yielding higher quantitative values in the network.

### GO Enrichment and Pathway Analysis

DAVID Bioinformatics Resources 6.8^14^ was used to perform GO enrichment analysis of the differentially expressed targets to explore their roles in many BPs. Twenty significantly enriched terms in the CC, BP, and MF categories are shown. ClueGO is a Cytoscape plugin for visualizing the non-redundant features of a large number of gene clusters in a functional grouping network to assess the enrichment of DCMPI candidate targets. The ClueGO network was created using kappa statistics, reflecting the relationship between terms based on the similarity of related genes. The significances of terms and groups are automatically calculated.

### Nuclear Staining With DAPI

HT-29 colon cancer cells were seeded into 24-well plates (5 × 10^4^ cells/well) and treated with 30 and 60 μg/mL DCMPI for 24 h. Then, the cells were rinsed with PBS, stained with DAPI (0.5 μg/mL) and further incubated for 20 min in the dark. The slides were observed under a fluorescence microscope (Nikon, Japan), and the cells with nucleus condensation or fragmentation were considered apoptotic.

### Apoptosis Assay With Annexin V-FITC/PI Staining

HT-29 colon cancer cells were seeded into 6-well plates (5 × 10^5^ cells/well) and cultured overnight prior to exposure to different concentrations of DCMPI (30 μg/mL, 60 μg/mL) for 24 h. Then, the percentage of apoptotic cells was determined by an Annexin V-FITC/PI staining kit following the manufacturer’s protocol. Cells were then analyzed with the Guava Easy cytometer (Merck Millipore Co. Ltd., Darmstadt, Germany) within 1 h.

### Mitochondrial Membrane Potential (Δψm) Measurements

Mitochondrial membrane potential (Δψm) was monitored utilizing the JC-1 cationic dye (Molecular Probes) as recommended by the manufacturer. HT-29 cells were seeded into 6-well plates (5 × 10^5^ cells/well) and cultured overnight prior to exposure to different concentrations of DCMPI (30 μg/mL, 60 μg/mL) for 24 h and then resuspended in a buffer solution containing 10 μg/mL JC-1 cationic dye. After 20 min of incubation in the dark at 37°C, the samples were rinsed with PBS to remove unreacted dye. The fluorescence intensity was read with the Guava Easy cytometer within 1 h.

### Intracellular ROS Measurement

The cell-permeable dye DCFH-DA (Molecular Probes) was used to assay intracellular ROS levels. This dye diffuses into cells and becomes trapped inside by de-esterification. After a reaction with peroxides, the fluorescent product, 5-chloromethyl-2′-7′-dichlorofluorescein (DCF), is formed. HT-29 cells were seeded into 6-well plates (5 × 10^5^ cells/well) and cultured overnight prior to exposure to different concentrations of DCMPI (30 μg/mL, 60 μg/mL) for 24 h and then resuspended in DMEM/F-12 medium containing 10 μM DCFH-DA. Thereafter, the samples were incubated in the dark at 37°C for 20 min and then rinsed with PBS to remove unreacted dye. The fluorescence intensity was read with the Guava Easy cytometer within 1 h.

### Apoptosis-Related Protein Assay

Protein extraction was performed as follows: after treatment with DCMPI (30 μg/mL, 60 μg/mL) for 24 h, HT-29 cells were cultured, collected, rinsed twice with ice-cold PBS, lysed, incubated in RIPA buffer containing a 1% protease inhibitor cocktail for 30 min on ice, and then centrifuged at 12,000 × *g* for 15 min. The supernatants were harvested and prepared by mixing with 5× sample buffer for subsequent Western blot analysis. The protein samples were then separated by 10% SDS-PAGE and transferred onto 0.22 μm polyvinylidene fluoride membranes (Millipore, Bedford, MA, United States). The membranes were blocked with 5% non-fat skim milk for 1 h at room temperature and then incubated with the appropriate primary antibodies overnight at 4°C. The membranes were incubated with a horseradish peroxidase-conjugated secondary antibody at room temperature for 2 h, followed by rinsing three times with 1× TBST. The protein bands were visualized utilizing ECL detection reagents (Bio-Rad, United States).

### Statistical Analysis

All data were analyzed with GraphPad Prism 5.0 software (GraphPad Software, Inc., La Jolla, CA, United States) and SPSS 20.0 Software (SPSS, Inc., Chicago, IL, United States). Furthermore, all statistical comparisons were performed using one-way analysis of variance followed by a *post hoc* Dunnett’s test. Values of *P* < 0.01 were considered statistically significant.

## Results

### Cell Cytotoxicity Assay of *P. igniarius* Extracts

**FIGURE 1:**
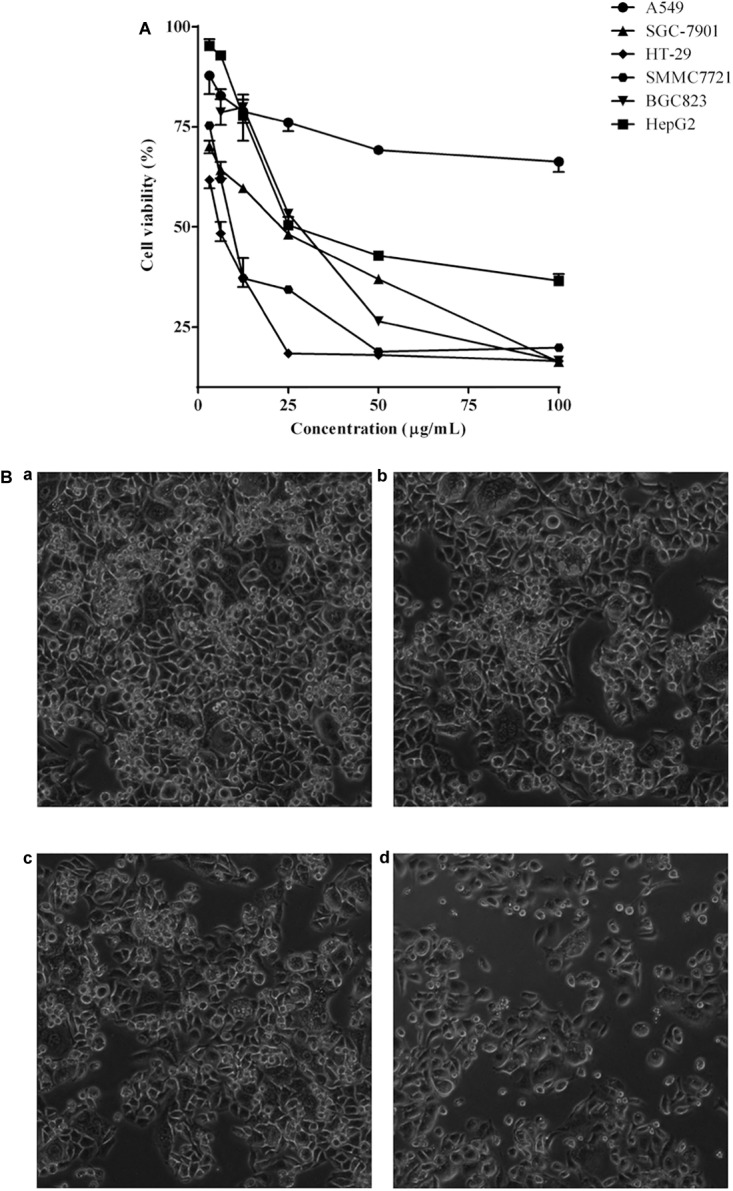
**(A)** Cytotoxic effects of DCMPI on the A549, SGC-7901, HT-29, SMMC7721, BGC-823 and HepG2 cell lines as determined by the MTT assay for 72 h. **(B)** Microscopic observation of HT-29 cells after administrating DCMPI for 72 h **(a)**: untreated; **(b)**: treated with 12.5 μg/mL DCMPI; **(c)**: treated with 25 μg/mL DCMPI; **(d)**: treated with 50 μg/mL DCMPI).

The cell viability inhibition induced by *P. igniarius* extracts, including the petroleum ether fraction, dichloromethane fraction, ethyl acetate fraction, *n*-butanol fraction and ethanol extract of *P. igniarius*, was evaluated *in vitro* with an MTT assay. Moreover, each cancer cell line was incubated with different concentrations of *P. igniarius* extracts for 72 h to compare their cytotoxicities. Notably, DCMPI as the strongest inhibitor decreased the viabilities of all the tested cell lines in a concentration-dependent manner, and especially significant cytotoxicity was observed in HT-29 cells (Figure 1A). Normal HT-29 cells appeared whole, healthy, and polygonal in shape under a microscope; however, after administering DCMPI, the HT-29 cells exhibited a number of morphological alterations, including cell shrinkage, condensed chromatin, membrane blebbing, and an increased density of apoptotic cells (Figure 1B).

### Characterization of DCMPI Chemical Constituents

**FIGURE 2:**
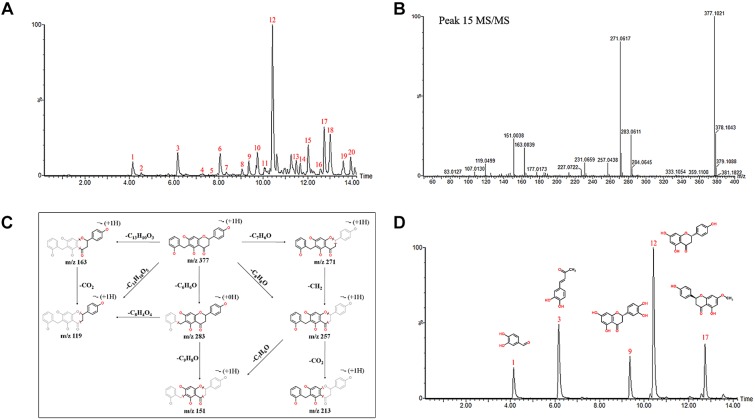
**(A)** UPLC-ESI-Q/TOF-MS BPI chromatogram of DCMPI. **(B)** Mass fragment spectrum of Phelligrin A in the negative ion mode. **(C)** Possible fragmentation mechanistic pathway of Phelligrin A. **(D)** Typical mixed standard substance chromatograms of protocatechuic aldehyde, osmundacetone, eriodictyol, naringenin, and sakuranetin.

The chemical profiles of DCMPI were analyzed by UPLC-ESI-Q/TOF-MS. All LC-MS data, including retention times, accurate molecular masses, and MS/MS data, are necessary for the structural analysis of compounds. The element compositions were calculated and clearly confirmed by combining with a mass accuracy (ppm) less than 5.0 using MarkerLynx (4.1) software. The majority of the components detected in the positive ion mode were also detected in the negative ion mode; therefore, data analysis was performed in only the negative ion mode. Using the optimal UPLC and Q/TOF-MS conditions described above, the BPI chromatogram was obtained in the negative ion mode (Figure 2A). A total of 20 peaks were obtained from the BPI chromatogram, and 19 of these peaks were identified or characterized (Table 1). Peak 15 was tentatively characterized as phelligrin A based on the mass spectrum and fragmentation pathway illustrated in Figure 2B,C.

**Table 1:** Characterization of chemical constituents of DCMPI by UPLC-ESI-Q/TOF-MS.

No.	RT (min)	Formula	Measured mass (m/z)	Theoretical mass (m/z)	Error (ppm)	Fragment ions	Identify
1	4.17	C_7_H_6_O_3_	137.0233	137.0239	−4.4	137[M-H]^−^, 108[M-H-CHO]^−^, 92[M-H-COOH]^−^	Protocatechuic aldehyde
2	4.55	C_9_H_6_O_4_	177.0182	177.0188	−4.5	177[M-H]^−^, 161[M-H-O]^−^, 135[M-H-COCH_2_]^−^	Esculetin
3	6.18	C_10_H_10_O_3_	177.0552	177.0552	0	177[M-H]^−^, 162[M-H-CH_3_]^−^, 134[M-H-COCH_3_]^−^	Osmundacetone
4	7.29	C_12_H_10_O_4_	217.0499	217.0511	−0.9	217[M-H]^−^, 177[M-H-C_3_H_4_]^−^, 161[M-H-C_3_H_4_O]^−^	Inotilone
5	7.53	C_41_H_72_O_10_	723.5004	723.4989	2.1	723[M-H]^−^, 677[M-H-COH-OH]^−^	Not identified
6	8.09	C_15_H_12_O_6_	287.0453	287.0556	−4.5	287[M-H]^−^, 259[M-H-CO]^−^, 243[M-H-COCH_3_]^−^, 152[M-H-C_8_H_7_O_2_]^−^, 125[M-H-C_9_H_6_O_3_]^−^	Dihydrokaempferol
7	8.39	C_25_H_20_O_9_	463.1023	463.1029	−1.3	463[M-H]^−^, 407[M-H-CH_3_-CO-CH]^−^, 379[M-H-CH_3_-CO-CH-CO]^−^, 243[M-H-C_6_H_5_O_2_-C_6_H_7_O_2_]^−^, 217[M-H-C_6_H_5_O_2_-C_7_H_5_O_3_]^−^, 159[M-H-C_6_H_5_O_2_-C_6_H_7_O_2_-COOH-CO]^−^, 135[M-H-C_6_H_5_O_2_-C_7_H_5_O_3_-C_5_H_6_O]^−^	Davallialactone
8	9.05	C_26_H_18_O_10_	489.0821	489.0822	−0.2	489[M-H]^−^, 445[M-H-CO_2_]^−^, 403[M-H-C_3_H_2_O_3_]^−^, 335[M-H-C_8_H_9_O_2_-OH]^−^, 241[M-H-C_8_H_9_O_2_-C_5_H_3_O_3_]^−^, 159[M-H-C_16_H_10_O_8_]^−^, 135[M-H-C_19_H_14_O_7_]^−^	Hypholomine B
9	9.37	C_15_H_12_O_6_	287.0554	287.0556	−0.7	287[M-H]^−^, 151[M-H-C_8_H_8_O_2_]^−^, 135[M-H-C_7_H_4_O_4_]^−^, 107[M-H-C_9_H_8_O_4_-]^−^	Eriodictyol
10	9.74	C_25_H_18_O_9_	461.0872	461.0873	−0.2	461[M-H]^−^, 377[M-H-C_4_H_4_O_2_]^−^, 333[M-H-C_5_H_4_O_4_]^−^, 257[M-H-C_11_H_8_O_4_]^−^, 241[M-H-C_11_H_8_O_5_]^−^, 159[M-H-C_15_H_10_O_7_]^−^, 134[M-H-C_17_H_11_O_7_]^−^	Epi-inoscavin A
11	10.12	C_25_H_18_O_9_	461.0869	461.0873	−0.9	461[M-H]^−^, 377[M-H-C_4_H_4_O_2_]^−^, 333[M-H-C_5_H_4_O_4_]^−^, 257[M-H-C_11_H_8_O_4_]^−^, 241[M-H-C_11_H_8_O_5_]^−^, 159[M-H-C_15_H_10_O_7_]^−^, 145[M-C_16_H_12_O_7_]^−^	Inoscavin A
12	10.42	C_15_H_12_O_5_	271.0607	271.0606	0.4	271[M-H]^−^, 177[M-H-C_6_H_6_O]^−^, 151[M-H-C_8_H_8_O]^−^, 119[M-H-C_7_H_4_O_4_]^−^, 107[M-H-C_9_H_8_O_3_]^−^	Naringenin
13	11.46	C_22_H_18_O_7_	393.0969	393.0974	−1.3	393[M-H]^−^, 365[M-H-CO]^−^, 299[M-H-C_6_H_6_O]^−^, 287[M-H-C_7_H_6_O]^−^, 259[M-H-C_8_H_6_O_2_]^−^, 243[M-H-C_8_H_6_O_3_]^−^, 231[M-C_9_H_6_O_3_]^−^	Oxyphelligrin A
14	11.65	C_16_H_14_O_6_	301.0704	301.0712	−2.7	301[M-H]^−^, 165[M-H-C_8_H_8_O_2_]^−^, 135[M-H-C_8_H_6_O_4_]^−^	Folerogenin
15	12.00	C_22_H_18_O_6_	377.1011	377.1025	−3.7	377[M-H]^−^, 283[M-H-C_6_H_6_O]^−^, 271[M-H-C_7_H_6_O]^−^, 257[M-H-C_8_H_8_O]^−^, 231[M-H-C_9_H_6_O_2_]^−^, 213[M-H-C_9_H_8_O_3_]^−^, 163[M-C_13_H_10_O_3_]^−^, 119[M-C_14_H_10_O_5_]^−^	Phelligrin A
16	12.53	C_13_H_18_O_3_	221.1174	221.1178	−1.8	221[M-H]^−^, 205[M-H-O]^−^, 177[M-H-C_2_H_4_O]^−^, 149[M-H-C_3_H_4_O_2_]^−^	Dehydrovomifoliol
17	12.7	C_16_H_14_O_5_	285.0751	285.0763	−4.2	285[M-H]^−^, 169[M-H-C_8_H_8_O]^−^, 119[M-H-C_9_H_10_O_3_]^−^	Sakuranetin
18	12.98	C_22_H_18_O_6_	377.1019	377.1025	−1.6	377[M-H]^−^, 283[M-H-C_6_H_6_O]^−^, 271[M-H-C_7_H_6_O]^−^, 257[M-H-C_8_H_8_O]^−^, 231[M-H-C_9_H_6_O_2_]^−^, 213[M-H-C_9_H_8_O_3_]^−^, 163[M-C_13_H_10_O_3_]^−^, 119[M-C_14_H_10_O_5_]^−^	Phelligrin B
19	13.56	C_23_H_20_O_6_	391.1174	391.1182	−2.0	391[M-H]^−^, 297[M-H-C_6_H_6_O]^−^, 285[M-H-C_7_H_6_O]^−^, 271[M-H-C_8_H_8_O]^−^, 245[M-H-C_9_H_6_O_2_]^−^, 165[M-H-C_15_H_14_O_2_]^−^, 119[M-C_15_H_12_O_5_]^−^, 93[M-C_17_H_14_O_5_]^−^	Methylphelligrin A
20	13.88	C_23_H_20_O_6_	391.1174	391.1182	−2.0	391[M-H]^−^, 297[M-H-C_6_H_6_O]^−^, 285[M-H-C_7_H_6_O]^−^, 271[M-H-C_8_H_8_O]^−^, 245[M-H-C_9_H_6_O_2_]^−^, 165[M-H-C_15_H_14_O_2_]^−^, 119[M-C_15_H_12_O_5_]^−^, 93[M-C_17_H_14_O_5_]^−^	Methylphelligrin B

**Table 2:** Linearity for the five ingredients using UPLC-ESI-Q/TOF-MS.

Compound name	RT (min)	Traces (m/z)	Calibration curves	Linear range (μg/mL)	*R*^2^
Protocatechuic aldehyde	4.14	137.02	*y* = 67.8718x + 451.627	4.61–73.15	0.9963
Osmundacetone	6.17	177.05	*y* = 188.218x + 930.296	3.97–63.44	0.9944
Eriodictyol	9.37	287.05	y = 84.2368 x–79.8782	3.91-62.50	0.9998
Naringenin	10.42	271.05	*y* = 353.18x + 1012.13	4.20–67.19	0.9961
Sakuranetin	12.73	285.07	*y* = 127.728–263.059	4.04–64.69	0.9985

### Quantification of Five Main DCMPI Ingredients

The established quantitative method was applied to determine the contents of protocatechuic aldehyde, osmundacetone, eriodictyol, naringenin, and sakuranetin in DCMPI. The linear parameters of the five ingredients are listed in Table 2. Typical chromatograms of the five ingredients are shown in Figure 2D. DCMPI contained approximately 1.17% protocatechuic aldehyde, 0.62% osmundacetone, 1.10% eriodictyol, 3.59% naringenin, and 3.71% sakuranetin.

### Putative DCMPI Target Prediction

**FIGURE 3:**
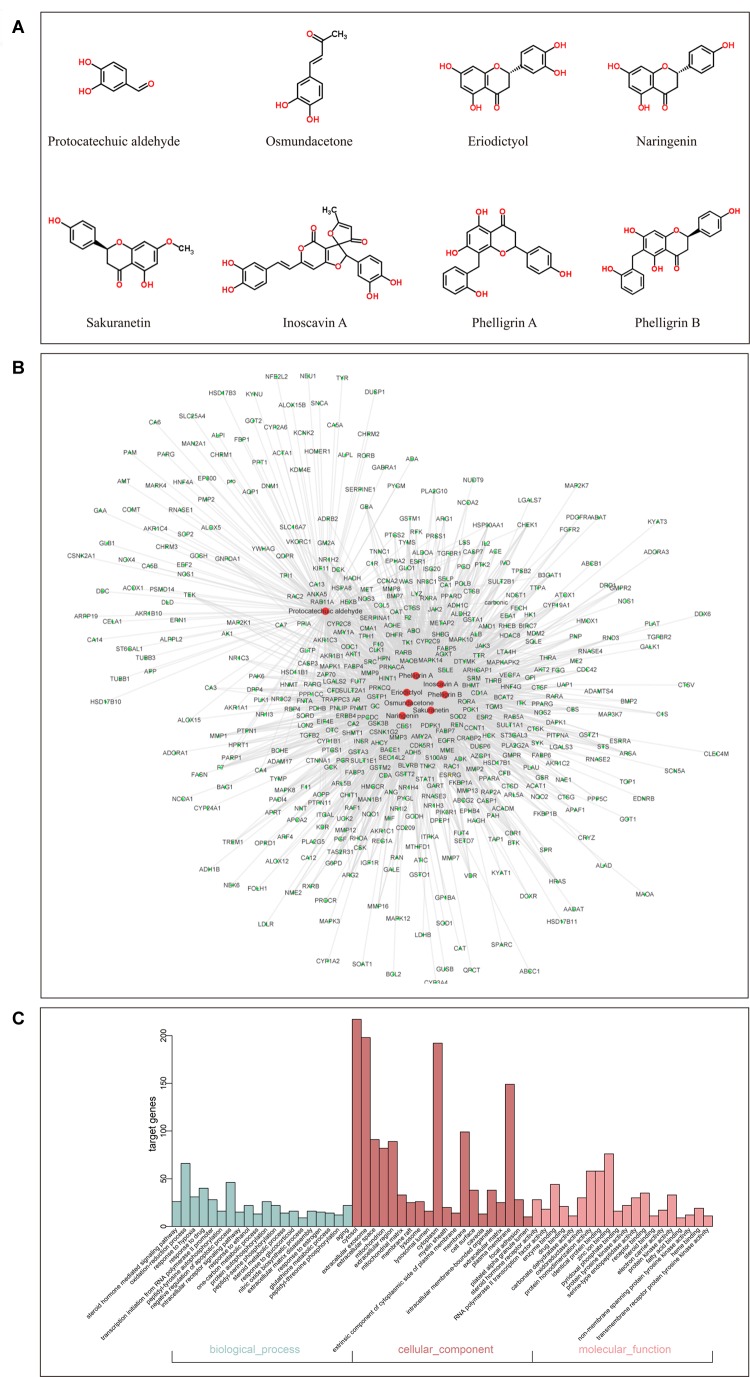
GO analysis of putative DCMPI targets and compound-target network construction. **(A)** Chemical structures of the eight main ingredients. **(B)** GO analysis of BP, cell component, and MF terms was performed on putative DCMPI targets, and the top 20 terms with *P* < 0.05 are shown. **(C)** The compound-target network was constructed by linking the 8 main compounds (red dots) and their potential targets (green dots).

By screening the eight main components of DCMPI (the structures are shown in Figure 3A) in the PharmMapper Server, SEA Search Server, STITCH and TCMSP databases, potential targets of DCMPI were obtained. A total of 1727 potential targets were predicted for the 8 main compounds (196 for protocatechuic aldehyde, 149 for osmundacetone, 284 for eriodictyol, 293 for naringenin, 285 for sakuranetin, 250 for inoscavin A, 270 for phelligrin A, and 264 for phelligrin B), and 473 targets remained after deleting duplicates.

### Compound-Target Network Construction and Analysis

Traditional Chinese medicine always exhibit versatile biological and pharmacological activities with complex chemical compositions by acting on multiple targets. Studying the complicated interactions between compounds and their targets at the system level may help comprehensively understand the mechanisms underlying TCM effects. We constructed a compound-target network based on the 8 candidate DCMPI compounds and their 473 potential targets. In total, 480 nodes and 1988 compound-target interactions are embodied in this network (Figure 3B).

### GO Enrichment and Pathway Analysis

GO analysis of the putative DCMPI targets was employed based on DAVID software for the visualization, annotation, and integrated discovery described by the BP, CC, and MF terms. In total, 671 BPs, 73 CCs, and 192 MFs that were enriched for this dataset were identified, of which 140 BPs, 55 CCs, and 140 MFs had *P*-values < 0.05. Figure 3C exhibits an overview of the GO analysis, and 20 remarkably enriched terms in the BP, CC, and MF categories are shown.

### Collection of Colon Cancer-Related Targets

**FIGURE 4:**
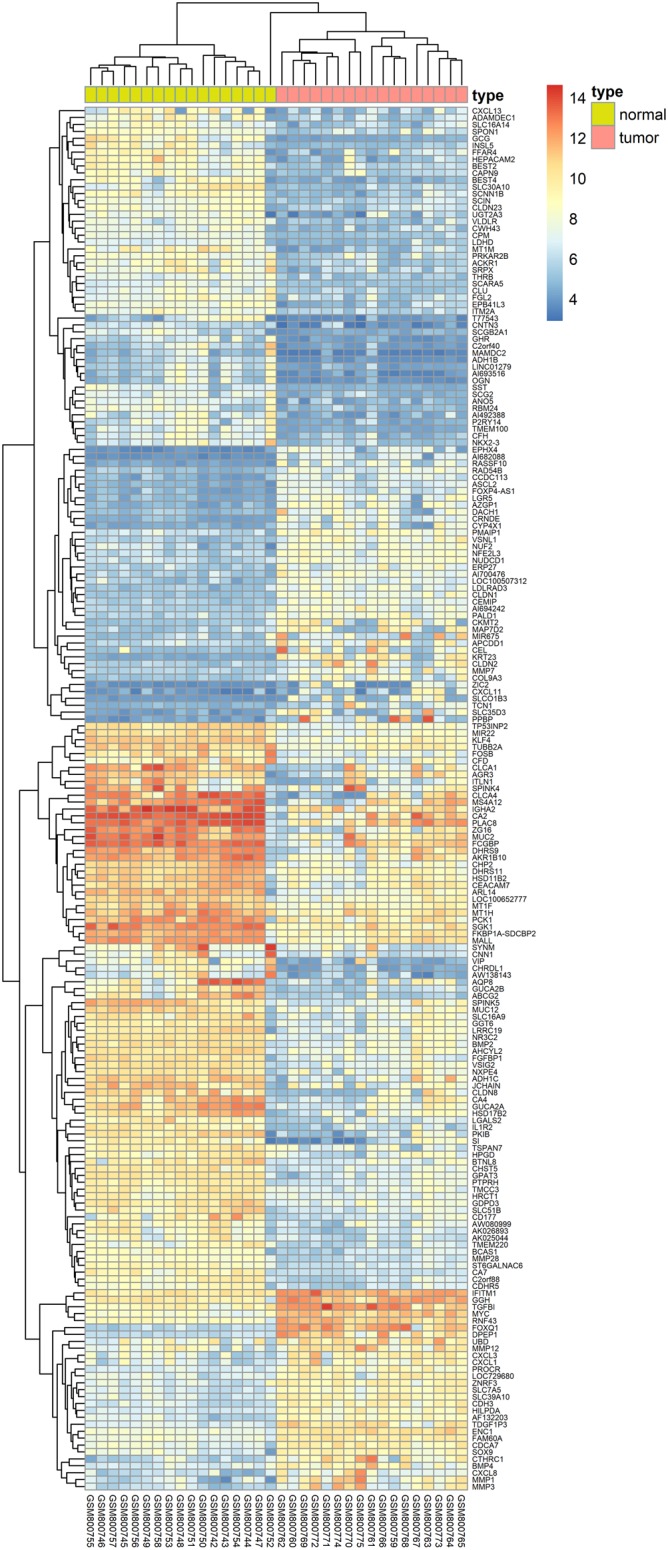
Identification of colon cancer-related targets using pre-existing microarray data. Two hundred DEGs identified by the limma package were highly related to colon cancer. *P* < 0.05 and FC > 2 were considered cut-off values.

We herein collected colon cancer-related targets from two main sources: DEGs obtained from publicly available microarray data and disease-related databases. As shown Figure 4, 200 DEGs were identified from the GEO repository microarray data, while other targets were obtained from five databases: 163 from GAD, 54 from OMIM, 25 from TTD, 66 from DrugBank, and 246 from CoolGen. After removing redundant genes, 349 colon cancer-related targets were collected. Of these, 84 genes were also the targets of DCMPI, which suggests an obvious therapeutic potential for *P. igniarius*.

### Identification of Candidate DCMPI Targets for Colon Cancer Treatment

**FIGURE 5:**
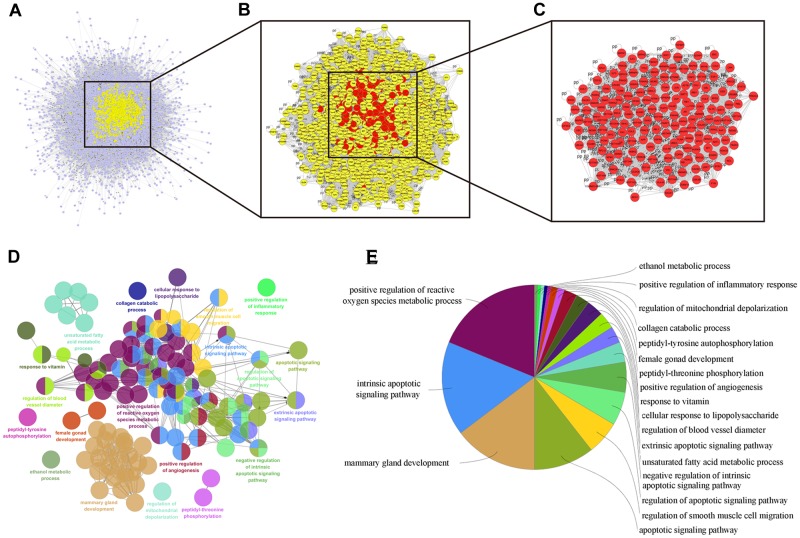
Candidate target identification and ClueGO pathway analysis. **(A)** Core CPPI network of DCMPI targets. **(B)** Large hubs of the DCMPI CPPI network extracted from **(A)** whose degrees were more than twice the median degree of all nodes in the network. **(C)** PPI network of the main DCMPI targets extracted from **(B)** constructed by calculating 6 topological features. **(D,E)** A functionally grouped network of enriched categories was generated for the target genes. GO terms are represented as nodes, and the node size represents the term’s enrichment significance. Functionally related groups partially overlap. Only the most significant term in the group is labeled. Representative enriched pathway (*P* < 0.05) interactions among the main DCMPI targets.

Numerous evidence in network biology has shown that genes and proteins exert their functions via interactions. Thus, we selected proteins as nodes for constructing the network. To elaborate the pharmacological mechanism by which DCMPI ameliorates colon cancer, we established PPI networks that may reflect the behavior and properties of biological molecules. First, a putative target PPI network of DCMPI-related genes was obtained using Cytoscape 3.2.1 software with the plugin BisoGenet (8933 nodes and 195,493 edges) and database retrieval of the PPI network of colon cancer-related targets (4615 nodes and 113,727 edges). We then merged these two networks to obtain a core PPI (CPPI) network that consisted of 4614 nodes and 113,727 edges. Subsequently, colon cancer targets of DCMPI were screened using the topological features of CPPI. Then, utilizing a Cytoscape plugin (CytoNCA), the main hubs of the network were screened by calculating the topological features for each hub, which were identified when their degree exceeded twice the median degree of all nodes in the network. A flow chart depicting the core target screening is presented in Figure 5A–C.

### Pathway Enrichment Analysis of DCMPI Targets

A Cytoscape plugin (ClueGO) was applied to further define the pathways involved in the biological networks identified above. As shown in Figure 5D,E, the biological networks consisted of 189 nodes and 1503 edges, and the potential targets were mainly assigned to positive regulation of ROS metabolic processes, intrinsic apoptotic pathways and mammary gland development. Of these, the first two pathways have well-established roles in cell apoptosis, and the results suggested that DCMPI is highly likely to exert its antitumor effect via the apoptotic pathway.

### Nuclear Staining With DAPI

**FIGURE 6:**
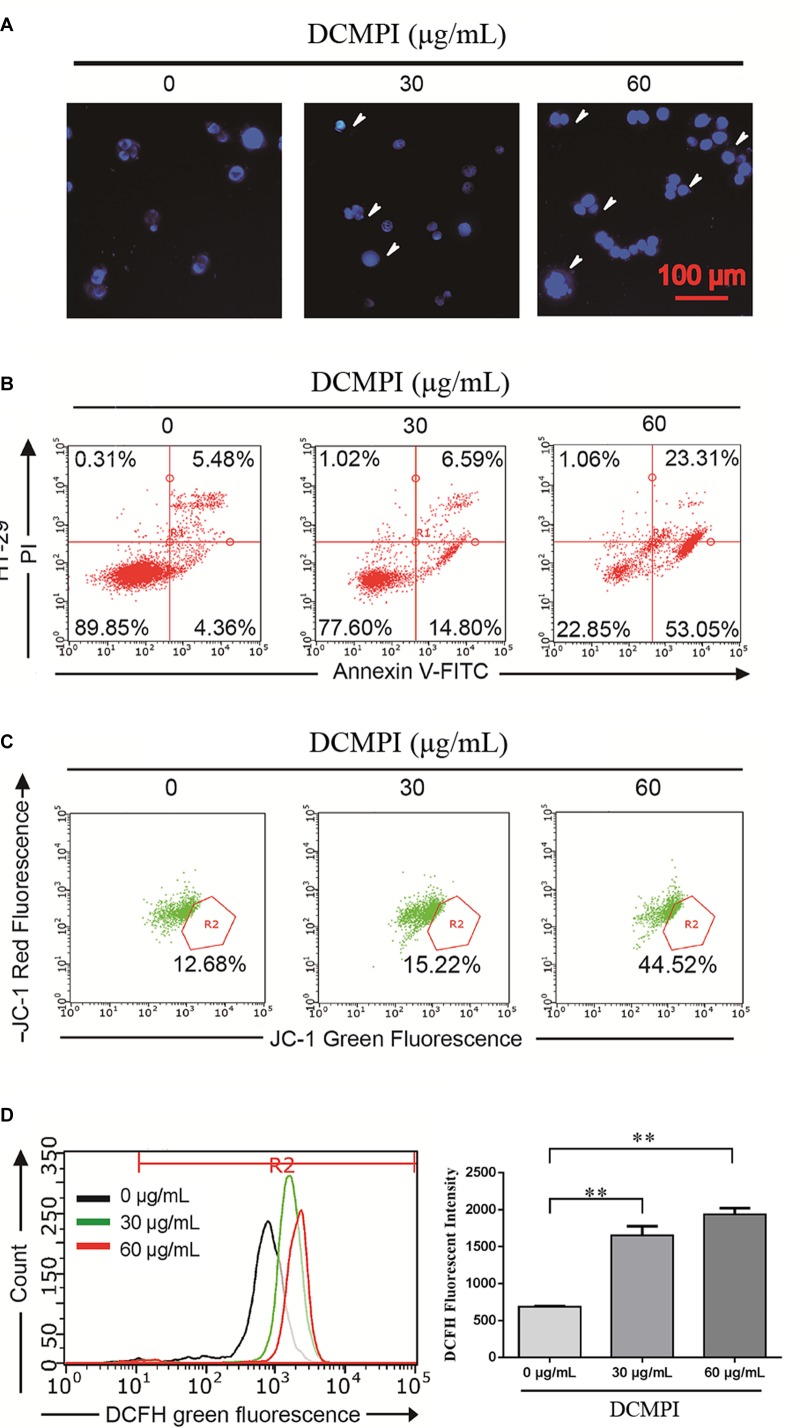
DCMPI induced apoptosis in HT-29 colon cancer cells. **(A)** Nuclear morphological changes were observed in DCMPI-exposed HT-29 cells by DAPI staining. **(B)** The percentage of apoptotic cells induced by DCMPI treatment was detected by Annexin V/PI double-staining analysis. **(C)** The effect of DCMPI on the mitochondrial membrane potential, which was determined using a JC-1 staining assay and flow cytometry. **(D)** The effect of DCMPI on the intracellular ROS, which was detected using DCFH-DA probes and flow cytometry. ^∗∗^*P* < 0.01. The data are presented as the mean ± SD from at least three independent experiments.

In this study, HT-29 cells were monitored by DAPI staining to determine the pro-apoptotic effect of DCMPI. The administration of DCMPI at doses of 30 and 60 μg/mL for 24 h dramatically induced nuclear morphological changes, such as apoptotic bodies and nuclear fragmentation, compared with the control group (Figure 6A).

### Apoptosis Assay With Annexin V-FITC/PI Staining

Next, the percentage of apoptotic cells induced by DCMPI treatment was detected by Annexin V-FITC/PI staining. As shown in Figure 6B, the numbers of HT-29 cells in both early- and late-stage apoptotic populations were remarkably increased in response to DCMPI (30 and 60 μg/mL). Therefore, this finding was consistent with the DAPI staining results and confirmed that DCMPI induced HT-29 cell apoptosis in a concentration-dependent manner.

### Mitochondrial Membrane Potential (MMP, Δψm) Assessment

The loss of Δψm, which occurs prior to caspase activation, is regarded as a hallmark of apoptosis. To determine whether DCMPI treatment activated the mitochondria-mediated apoptotic pathway, we utilized the JC-1 fluorescent probe to evaluate alterations in Δψm by flow cytometry. In apoptotic cells with low Δψm, JC-1 remains in the monomeric form, and the loss of Δψm is followed by a red-to-green shift. The percentage of green JC-1 was significantly elevated by treatment with DCMPI (Figure 6C), and DCMPI thus markedly increased the mitochondrial membrane permeability and induced Δψm collapse.

### Intracellular ROS Detection

Reactive oxygen species generation was closely associated with the cancer cell death caused by mitochondrial dysfunction and subsequent mitochondria-induced apoptosis. To identify the roles of ROS in DCMPI-induced colon cancer cell death, we measured intracellular ROS production by DCFH-DA staining with flow cytometry. DCMPI significantly increased the accumulation of fluorescent dye in HT-29 cells in a concentration-dependent manner (Figure 6D).

### Apoptosis-Related Protein Assay

**FIGURE 7:**
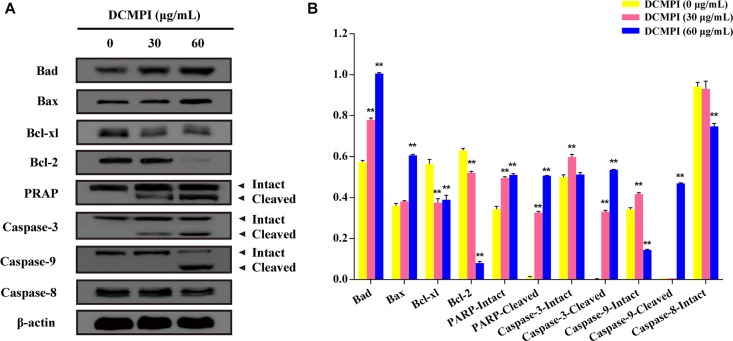
Western blot analysis of apoptosis-related protein expression. **(A)** Bad, Bax, Bcl-xl, Bcl-2, PARP, caspase-3, caspase-9, and caspase-8 protein expression in HT-29 cells after DCMPI treatment (30 and 60 μg/mL) for 24 h. **(B)** Densitometric analysis of Bad, Bax, Bcl-xl, Bcl-2, PARP, caspase-3, caspase-9, and caspase-8 expression. ^∗∗^*P* < 0.01. The data are presented as the mean ± SD from at least three independent experiments.

DCMPI not only significantly up-regulated the protein expression of Bax and Bad but also simultaneously attenuated the protein levels of Bcl-2 and Bcl-xl. These results revealed that DCMPI alters the Bax/Bcl-2 ratio in HT-29 cells, which subsequently stimulates the mitochondrial-mediated apoptosis pathway. Furthermore, DCMPI (30 and 60 μg/mL) induced both PARP and caspase-3/9 cleavage to aggravate HT-29 cell apoptosis (Figure 7).

**FIGURE 8:**
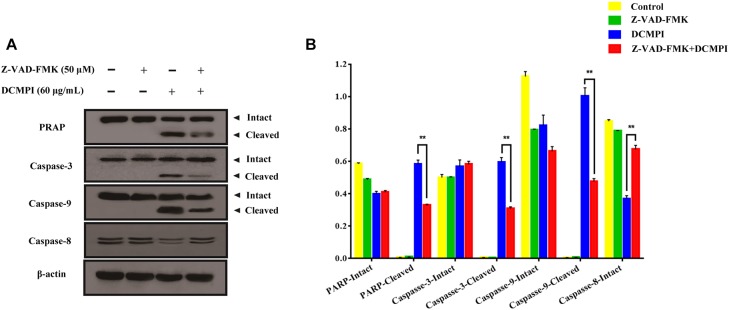
The effect of Z-VAD-FMK (50 μM) pretreatment and DCMPI (60 μg/mL) treatment on the cleavage of PARP and caspase-3/9 in HT-29 cells. **(A)** PARP, caspase-3, caspase-9, and caspase-8 protein expression in HT-29 cells after treatment for 24 h. **(B)** Densitometric analysis of PARP, caspase-3, caspase-9, and caspase-8 expression. ^∗∗^*P* < 0.01. The data are presented as the mean ± SD from at least three independent experiments.

To clarify whether apoptosis is initiated by caspase cascade activation, we adopted the caspase inhibitor Z-VAD-FMK. Western blot analysis revealed that the cleavage of PARP and caspase 3/9 caused by DCMPI was dramatically inhibited by Z-VAD-FMK (50 μM) pretreatment (Figure 8). Together, these results clearly support that DCMPI induces HT-29 cell apoptosis via the mitochondrial apoptosis pathway.

## Discussion

Recently, medicinal fungi were shown to be ubiquitously present in nature, recognized as a promising source of food and medicine with multi-targeted effects and to have low toxicity (Gargano et al., 2017). Intriguingly, numerous preclinical and clinical trials have demonstrated that medicinal fungi contain a variety of structurally unique metabolites, which most likely possess significant activities like anti-inflammatory and antitumor (Piska et al., 2017). For instance, *P. igniarius* has been proposed as a prevalent type of edible and medicinal mushroom that has multiple highly effective protective properties (Dong et al., 2016). More importantly, *P. igniarius* attracts overwhelming attention as a potential candidate for anticancer therapeutics (De Silva et al., 2012). Cancer is a complex disease and tumorigenesis is related to inflammation (Guo et al., 2017). *P. igniarius* has potential anti-inflammatory and antitumor activities, hopeful to be developed a functional food for the prevention and control of inflammation-induced tumorigenesis in the future. Previous studies had established that the ethanol extract of *P. igniarius* suppresses SK-Hep-1 and RHE cell proliferation in a dose-dependent manner (Song et al., 2008). Furthermore, the hot water extract of *P. igniarius* directly inhibits S180 tumors and simultaneously exhibits an immune-regulatory effect on S180-bearing mice (Yang et al., 2006). However, to date, these studies largely emphasize the pharmacological effects of *P. igniarius* extracts, and no studies have characterized the chemical composition *P. igniarius* extracts or clarified their underlying mechanisms.

Traditional Chinese medicine network pharmacology was a new research strategy for translating TCM from an experience-based medicine to an evidence-based medicine system, which predicted the target profiles and pharmacological actions of herbal compounds, and revealed drug-gene-disease co-module associations to interpret the combinatorial rules and network regulation effects of herbal formulae (Liang et al., 2014; Li and Zhang, 2013). It provided a new paradigm for revealing the pharmacodynamics substance basis and mechanisms of TCM and revealing the problem of the effectiveness of TCM (Wang et al., 2017).

Therefore, we herein utilized a combination method that included a cytotoxicity test, phytochemical analysis, TCM network pharmacology, and cell and molecular biology experiments to elucidate the antitumor substances and potential mechanisms of *P. igniarius*. First, cytotoxicity experiments were used to show that DCMPI had a certain inhibitory effect on each tumor strain. Among the strains, the inhibitory effects on the BGC-823, SMMC7721 and HT-29 cell lines were higher than those on the others. Second, we elucidated the systemic phytochemical composition of DCMPI via UPLC-ESI-Q/TOF-MS. As previously described, 19 constituents were identified or tentatively characterized, and 5 of them were quantified by standard substances. Numerous ingredients in DCMPI have been reported to possess antitumor activity or cytotoxicity. For example, naringenin, a flavanone compound, has highly effective, diverse bioactivities, including anticancer activity (Chang et al., 2017). Eriodictyol also exerts multiple bioactive effects, and it especially inhibits cell proliferation and transformation (Zhu et al., 2015). Dihydrokaempferol serves as a flavonol extensively in citrus fruits and provides anticancer effects via suppressing cell migration and invasion (Liu X. et al., 2017). The flavonoids phelligrin A, phelligrin B, methylphelligrin A and methylphelligrin B were first discovered in the *Phellinus* family and shown to possess multiple bioactivities (Wu et al., 2011). Furthermore, several studies revealed that phelligrin A and phelligrin B provide selective cytotoxicity against cancer cells (Lee and Yun, 2011). In summary, the main compounds in DCMPI are attributed to the flavone family, and most have anticancer activity and likely account for the antitumor activity of *P. igniarius.*

Because of their complex compositions and mechanisms of action, investigating the pharmacology of TCM is a daunting task. Here, to explore the antitumor mechanism of DCMPI, we selected eight major components of DCMPI and utilized TCM network pharmacology approach to clarify their potential mechanisms. The network pharmacological analysis showed that DCMPI exerted its anticolon cancer function mainly via the positive regulation of ROS metabolic processes and intrinsic apoptotic pathways.

Apoptosis induction has been recognized as an essential mechanism in antitumor therapeutics (Janssen, 2001). Notably, numerous studies have demonstrated that anticancer agents exert their anti-proliferative effects mainly via two different apoptosis pathways involving mitochondria or death receptors (Liu B. et al., 2017). In particular, the mitochondria-associated pathway is a classic intrinsic pathway that is initiated by ROS overproduction, resulting in the depletion of Δψm (Kuranaga, 2012). In general, a variety of protein molecules are involved in regulating the mitochondrial apoptotic pathway, including pro-apoptotic members (e.g., Bax and Bad) and anti-apoptotic members (e.g., Bcl-2 and Bcl-xl) (Czabotar et al., 2014). Furthermore, the pathway activates the specific pivotal proteinases as initiator caspase-9 and effector caspase-3, which subsequently leads to DNA fragmentation and nuclear PARP degradation during apoptosis (Kuranaga, 2012).

To verify the network pharmacology prediction results, *in vitro* cell experiments were carried out. DAPI and Annexin V-FITC/PI staining were performed to determine the apoptotic alterations associated with the cytotoxicity of HT-29 cells. Apoptotic bodies, nuclear fragmentation and early- and late-stage apoptotic populations were increased by DCMPI. Therefore, the cytotoxic effect of DCMPI on HT-29 cells appeared to be mediated by apoptosis. Apoptosis is considered to be a regulated form of cell death, and dysregulated apoptosis leads to a variety of aberrant pathological conditions and subsequent diseases, especially cancer (Sankari et al., 2012; Goldar et al., 2015). Remarkably, two main signaling pathways involving caspase-mediated apoptosis have been identified: the mitochondrial-mediated intrinsic pathway and the death receptor-mediated extrinsic pathway (Khan et al., 2014). Compelling evidence has indicated that increased levels of intracellular ROS result in mitochondrial swelling and MMP loss, which, in turn, activates the intrinsic apoptotic pathway. Because ROS overproduction not only causes severe DNA damage but also suppresses tumor growth, aggravated cell structures break down, and cancer cells die (Renschler, 2004). In this study, DCMPI stimulated the generation of intracellular ROS in a dose-dependent manner, induced MMP collapse and ultimately contributed to mitochondria-mediated apoptosis.

**FIGURE 9:**
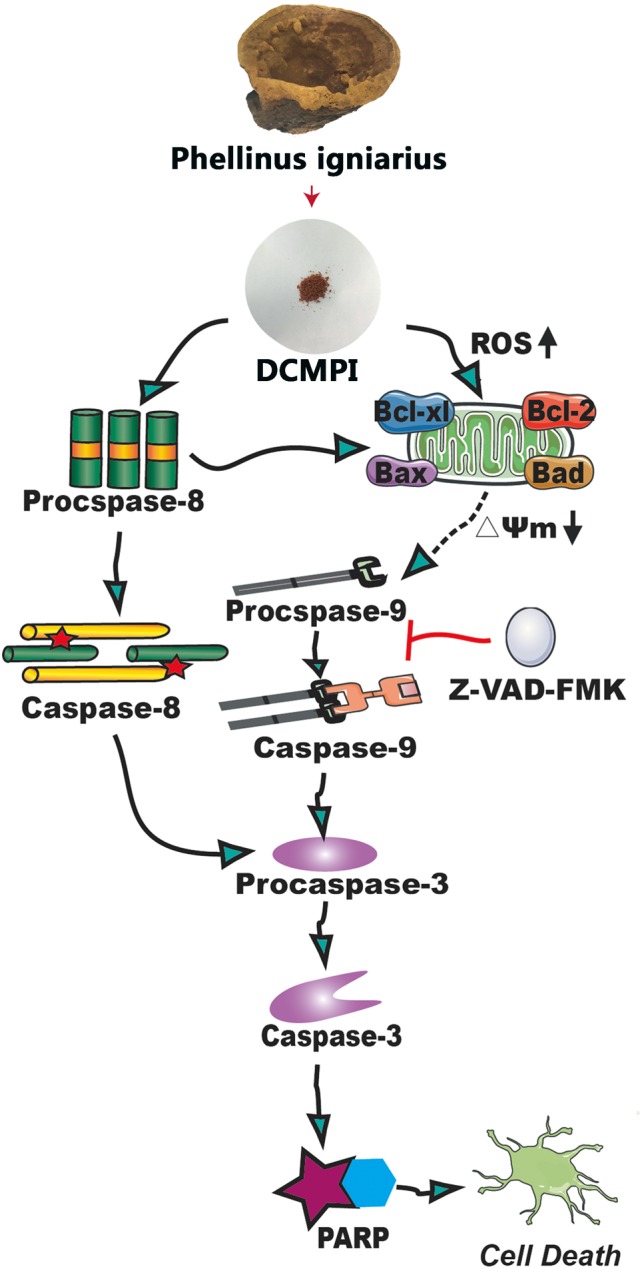
Molecular mechanisms underlying the antitumor activity of DCMPI. DCMPI increased intracellular ROS levels and induced HT-29 cell apoptosis mainly mediated via the mitochondrial apoptosis pathway.

Bcl-2 family members, including Bcl-2, Bcl-xl, Bax, and Bad, are major regulators located in the mitochondria (Borkan, 2016). As anti-apoptotic proteins, Bcl-2 and Bcl-xl intervene in various apoptotic signals, whereas Bax and Bad serve as pro-apoptotic proteins and trigger the activation of caspases by mitochondrial dysfunction. In the present study, DCMPI down-regulated anti-apoptotic Bcl-xl and Bcl-2 expression and simultaneously enhanced the translocation of the activated pro-apoptotic proteins Bax and Bad. More importantly, subsequent activation of the caspase-dependent pathway was recognized as a pivotal step in the apoptotic process, and a series of initiator caspases was involved in the progression. Briefly, activation of the initiator caspase as caspase-9 activates downstream or executioner caspases undergoing apoptosis, such as caspase-3 (Nuñez et al., 1998; Kim and Hong, 2011). Furthermore, executioner caspase activation was responsible for a series of apoptotic biochemical characteristics, especially the cleavage of PARP, which facilitated cellular disassembly and resulted in DNA fragmentation. Herein, our findings demonstrated that DCMPI induced apoptosis in HT-29 cells by up-regulating cleaved caspase-3/9 and PARP. In addition, pretreatment with the pan-caspase inhibitor Z-VAD-FMK inhibited caspase and PARP cleavage, which confirmed that the pro-apoptotic effect of DCMPI in HT-29 cells was exerted via a caspase-dependent pathway (Figure 9).

## Conclusion

In the current study, we first demonstrated that the DCMPI dramatically suppressed HT-29 colon cancer cell proliferation. Then, we characterized 19 main constituents of DCMPI by UPLC-ESI-Q/TOF-MS, and quantified 5 of them. Furthermore, network pharmacology analysis focusing on the 8 high content compounds of DCMPI (different from other fractions) was performed, revealing that the potential targets were mainly associated with the positive regulation of ROS metabolic processes and intrinsic apoptotic pathways. Finally, cellular tests verified that DCMPI increased intracellular ROS levels to induce HT-29 cell apoptosis. Taken together, our results provide the antitumor chemical composition of and mechanistic insights into *P. igniarius*, which may be exploited as a promising therapeutic option for colon cancer.

## Author Contributions

YD and PQ wrote the manuscript. YD, PQ, RZ, LZ, PZ, and YW conducted the research and analyzed the data. CL, KC, DS, and HZ conceived or designed the studies. All authors read and approved the final manuscript.

## Conflict of Interest Statement

The authors declare that the research was conducted in the absence of any commercial or financial relationships that could be construed as a potential conflict of interest. The reviewer YZhu declared a shared affiliation, with no collaboration, with several of the authors to the handling Editor at the time of review.

## Footnotes

1 http://www.ncbi.nlm.nih.gov/pubmed

2 http://www.chemspider.com/

3 http://www.hmdb.ca/

4 http://metlin.scripps.edu

5 https://omictools.com/pharmmapper-tool

6 http://sea.bkslab.org/

7 http://stitch.embl.de/

8 http://lsp.nwu.edu.cn/tcmspsearch.php

9 http://www.ncbi.nlm.nih.gov/geo/

10 http://www.omim.org/

11 https://db.idrblab.org/ttd/

12 http://www.drugbank.ca/

13 http://ci.smu.edu.cn/CooLGeN/Home.php

14 https://david.ncifcrf.gov/

## Abbreviations

BP: biological process
BPI: base peak intensity
CC: cellular component
DAPI: 4’,6-diamidino-2-phenylindole
DEGs: differentially expressed genes
DMSO: dimethyl sulfoxide
ECL: enhanced chemiluminescence
GAD: genetic association database
GEO: gene expression omnibus database
GO: gene ontology
HMDB: Human Metablome Database
MF: molecular function
MMP: mitochondrial membrane potential
MTT: 3-[4,5-dimethylthiazol-2-yl]-2,5-diphenyltetrazolium bromide
OMIM: online mendelian inheritance in man
PARP: poly ADP-ribose polymerase
PBS: phosphate buffer saline
*P. igniarius*: *Phellinus igniarius*
*P. igniarius* DCM: dichloromethane extract of *Phellinus igniarius*
PPI: protein–protein interaction
ROS: reactive oxygen species
SDS-PAGE: sodium dodecyl sulfate polyacrylamide gel electrophoresis
SEA: similarity ensemble approach
TCM: traditional Chinese medicine
TCMSP: traditional Chinese medicine systems pharmacology
TTD: therapeutic target database
UPLC-ESI-Q/TOF-MS: ultra performance liquid chromatography-electrospray ionization- quadrupole/time-of-flight mass spectrometry
